# Collateralization of cerebellar output to functionally distinct brainstem areas. A retrograde, non-fluorescent tracing study in the rat

**DOI:** 10.3389/fnsys.2014.00023

**Published:** 2014-02-21

**Authors:** Tom J. H. Ruigrok, Thea M. Teune

**Affiliations:** Department of Neuroscience, Erasmus MC RotterdamRotterdam, Netherlands

**Keywords:** cerebellar nuclei, red nucleus, inferior olive, nucleus reticularis tegmenti pontis, nucleus of Darkschewitsch

## Abstract

The organization of the cerebellum is characterized by a number of longitudinally organized connection patterns that consist of matching olivo-cortico-nuclear zones. These entities, referred to as modules, have been suggested to act as functional units. The various parts of the cerebellar nuclei (CN) constitute the output of these modules. We have studied to what extent divergent and convergent patterns in the output of the modules to four, functionally distinct brain areas can be recognized. Two retrograde tracers were injected in various combinations of the following nuclei: the red nucleus (RN), as a main premotor nucleus; the prerubral area, as a main supplier of afferents to the inferior olive (IO); the nucleus reticularis tegmenti pontis (NRTP), as a main source of cerebellar mossy fibers; and the IO, as the source of climbing fibers. For all six potential combinations three cases were examined. All nine cases with combinations that involved the IO did not, or hardly, resulted in double labeled neurons. In contrast, all other combinations resulted in at least 10% and up to 67% of double labeled neurons in cerebellar nuclear areas where both tracers were found. These results show that the cerebellar nuclear neurons that terminate within the studied areas represent basically two intermingled populations of projection cells. One population corresponds to the small nucleo-olivary neurons whereas the other consists of medium- to large-sized neurons which are likely to distribute their axons to several other areas. Despite some consistent differences between the output patterns of individual modules we propose that modular cerebellar output to premotor areas such as the RN provides simultaneous feedback to both the mossy fiber and the climbing fiber system and acts in concert with a designated GABAergic nucleo-olivary circuit. These features seem to form a basic characteristic of cerebellar operation.

## Introduction

The cerebellum provides its regulatory influence on many aspects of the central nervous system through its cerebellar nuclear output. It has become abundantly clear that cerebellar nuclear efferents terminate, mostly contralaterally, in a great variety of brain stem regions. Ramón y Cajal (1911) already described that, immediately after its decussation in the midbrain, the superior cerebellar peduncle (scp) branches into ascending and descending bundles (also see Voogd and Van Baarsen, 2014). The descending branch is located in the ventromedial tegmentum and mainly terminates in the nucleus reticularis tegmenti pontis (NRTP), the basilar pontine nuclei, the pontine, and medullary reticular formation and in the inferior olive (IO). The ascending branch terminates in many areas such as the red nucleus (RN), periaqueductal gray, the prerubral area (including the area surrounding the fasciculus retroflexus, PreRN), the accessory oculomotor nuclei, the superior colliculus, zona incerta, and the thalamus (Chan-Palay, 1977; Faull, 1978; Faull and Carman, 1978; Teune et al., 2000). Since the cerebellar nuclei (CN) serve as the output stations of the cerebellar modules as defined by the organization of their cortico-nuclear and olivo-cortical connections (Voogd and Bigaré, 1980; Buisseret-Delmas and Angaut, 1993; Apps and Garwicz, 2005; Ruigrok, 2011), it has become a point of interest to investigate in what way this modular organization becomes implemented within the brain stem circuitry. More detailed knowledge on the distribution of information processed by cerebellar modules has become even more pressing since behavioral studies indeed suggest that cerebellar modules may represent functional entities (Godschalk et al., 1994; Van Der Steen et al., 1994; Apps and Garwicz, 2005; Pijpers et al., 2008; Cerminara and Apps, 2011).

Within the CN, at least two types of projection cells can be distinguished. One type consists of relatively small cells that are GABAergic and predominantly, if not exclusively, project to the IO (Chan-Palay, 1977; Bentivoglio and Kuypers, 1982; Buisseret-Delmas and Angaut, 1989; De Zeeuw et al., 1989; Fredette and Mugnaini, 1991; Teune et al., 1995) whereas the other group consists of large- and medium-sized cells that are mostly excitatory (Toyama et al., 1968) although recently a separate glycinergic group of large projection neurons has been distinguished in the medial cerebellar nucleus (MCN) (Bagnall et al., 2009). Neurons that belong to the population of large projection cells may collateralize to a wide array of target sites as has been shown with fluorescent retrograde double-labeling techniques. In the rat, collateralization has been demonstrated for combinations involving the medullary and pontine reticular formation (or the spinal cord), the thalamus, the superior colliculus, the accessory oculomotor nuclei or the basilar pontine nuclei (Bentivoglio and Kuypers, 1982; Gonzalo-Ruiz and Leichnetz, 1987; Lee et al., 1989). The group of medium- and large-sized relay cells does not appear to terminate within the IO (Bentivoglio and Kuypers, 1982; Lee et al., 1989; Teune et al., 1995). Also, although electrophysiological data, performed in the cat, have suggested otherwise (Ban and Ohno, 1977; McCrea et al., 1978; Tolbert et al., 1978), it seems unlikely that in the rat the nucleo-olivary pathway contributes to the crossed ascending branch of the scp. Indeed, double-labeling experiments with combinations of the RN and IO (Teune et al., 1995) or of thalamus or superior colliculus and the caudal ventral medulla (Bentivoglio and Kuypers, 1982) failed to show double-labeled small neurons in the CN.

In the present study, we make use of small, confined injections of highly selective and permanently visible, retrogradely transported tracers (Ruigrok and Apps, 2007) in order to further investigate the degree of collateralization of individual neurons within the CN. Since, as outlined above, the output of individual CN may reflect functional characteristics, particular interest was paid to the potential divergence of this output to four well-known, but functionally rather different cerebellar target sites. We have selected the RN as an example of a premotor area (Ruigrok, 2004); the IO as the sole source of cerebellar climbing fibers (Desclin, 1974); the NRTP as an important mossy fiber source which also collateralizes to the CN (Mihailoff, 1993; Ruigrok, 2004), and the prerubral area (PreRN), here defined as including the region surrounding the fasciculus retroflexus from which a major projection to the IO originates (De Zeeuw and Ruigrok, 1994; Ruigrok, 2004; Onodera and Hicks, 2009). The results will be discussed in relation to the coupling of the motor output of the cerebellum with recurrent mossy fiber and climbing fiber paths. Differences in collateralization between different parts of the CN will be discussed in relation to the modular organization of the cerebellum (Ruigrok, 2011).

## Materials and methods

### Surgical procedures and tracer application

All experiments were performed on purpose-bred male Wistar rats. The experimental procedure fully adhered to EC guidelines and was accorded by a national ethical committee overseeing animal experiments (DEC). A total of 18 rats, weighing 250–300 grams, were anesthetized by an intraperitoneal injection with a cocktail of ketamine (80 mg/kg) and xylazine (5 mg/kg) and were subsequently mounted in a stereotactic device according to Paxinos and Watson (2005). Additional dosages of anesthetics were given when needed.

Access to the cerebellum and lower brain stem was gained by partially removing the squamosal part of the occipital bone after partition of the covering skin and muscles through a midline incision over the skull and neck area. The IO was reached by penetrating the medulla oblongata at obex level dorsally, at an angle of 45° with the horizontal plane. The RN and PreRN were approached through a small hole drilled in the parietal bone directly overlying these areas (between 3.0 and 5.0 mm rostral to the interaural line, laterality: 0.8–1.0 mm). The NRTP was approached stereotactically through the caudal cerebellum (entering between lobule IXb and IXc) at an angle of 34° with the horizontal plane. In this way, the electrode would pass ventrally to the CN, avoiding possible spread of tracer into the CN and/or IO.

All injections were made at the left side of the brainstem using double barrel glass micro pipettes (overall tip diameter: 12–18 μm). One barrel contained the tracer, whereas the other was filled with 4 M NaCl and was connected to standard electrophysiological equipment. In this way, and prior to the actual application of tracers, electrophysiological recordings were made in order to verify the location of the glass electrode tip. The RN and IO both demonstrate a spontaneous and characteristic firing pattern (Gellman et al., 1983; Ruigrok and Voogd, 1995). The location of the NRTP was inferred by identifying the medial lemniscus, which demonstrated increased firing rates in response to tapping or touching the animal's contralateral body, and positioning the electrode tip slightly dorsal to this region.

As retrograde tracers, cholera toxin ß subunit (CTb: 1% w/v in 0.1 M phosphate buffer, pH 7.2; List Biol. Lab. Inc. Campbell, CA) was used in combination with a gold-lectin conjugate, wheatgerm agglutinin coupled to bovine serum albumin and 10 nm gold sol particles (Ruigrok and Apps, 2007). CTb was delivered iontophoretically by means of a 4 μ A positive current for 10–15 min., with a 7 s on/off cycle. The gold-lectin injections were delivered using a home-made pressure device and comprised approximately 30 nl for PreRN and RN, 50 nl for the NRTP and up to around 100 nl using several tracks for the IO (Ruigrok and Apps, 2007).

In order to reduce potential inadvertent labeling of fibers of passage (Chen and Aston-Jones, 1995), CTb was preferentially injected into the more distal of the injection sites, whereas gold-lectin, which has not been reported to be taken up by passing fibers (Menetrey, 1985; Llewellyn-Smith et al., 1992; Ruigrok and Apps, 2007) was predominantly selected for injections more proximal to the CN. However, when occasionally tracer injections were reversed this did not appear to influence the resulting labeling (Table 1).

**Table 1:** **Numbers of single and double-labeled neurons plotted in 5 equidistant sections of the CN of all studied cases**.

**Exp. #**	**Combination**		**Total # of cells plotted in CN**			**# of plotted cells in overlapping areas**		
	**Injected**	**Tracer**	**# cells labeled from 1**	**# cells labeled from 2**	**# cells labeled from 1 and 2**	**# cells labeled from 1**	**# cells labeled from 2**	**# cells labeled from 1 and 2**
	**1: IO**	**2: RN**						
T45	Gold-lectin	CTb	527	463	2	501	340	2
T42	Gold-lectin	CTb	698	423	0	460	380	0
T39	Gold-lectin	CTb	491	379	1	433	208	1
	**1: IO**	**2: PreRN**						
T53	CTb	Gold-lectin	474	240	0	336	217	0
T54	CTb	Gold-lectin	503	282	0	338	247	0
T55	Gold-lectin	CTb	827	237	1	584	217	1
	**1: IO**	**2: NRTP**						
T61	CTb	Gold-lectin	511	293	3	196	187	3
T58	CTb	Gold-lectin	229	161	2	70	99	2
T68	CTb	Gold-lectin	167	513	2	149	213	2
	**1: NRTP**	**2: RN**						
T56	Gold-lectin	CTb	615	460	225	568	407	225
T60	Gold-lectin	CTb	375	493	78	250	341	78
T64	Gold-lectin	CTb	399	779	180	392	557	180
	**1: NRTP**	**2: PreRN**						
T63	Gold-lectin	CTb	521	425	126	508	387	126
T65	Gold-lectin	CTb	315	862	140	482	298	140
T69	Gold-lectin	CTb	322	149	33	312	99	33
	**1: RN**	**2: PreRN**						
T118	Gold-lectin	CTb	967	106	71	679	105	71
R746	Gold-lectin	CTb	543	287	86	495	233	86
R747	Gold-lectin	CTb	582	452	176	582	350	176

*The total number (#) of plotted neurons is given as well as the number of labeled neurons that were located in the same (i.e., “overlapping”) area*.

After injection, all wounds were sutured and the animals recovered uneventfully and survived for 4 or 5 days during which they did not demonstrate signs of stress and/or discomfort.

### Perfusion and immunohistochemistry

Before perfusion, the animals were deeply re-anaesthetized with an overdose of sodium pentobarbital (120 mg/kg i.p.), and transcardially perfusion-fixed by first clearing the cardiovascular system with a rinsing solution (300 ml of a 0.8% NaCl, 0.8% sucrose, 0.4% d-glucose solution in 0.05 M phosphate buffer, PB, pH 7.2, at room temperature: RT), followed by fixative (1000 ml of a freshly prepared solution containing 4% paraformaldehyde, 4% sucrose, 0.1% glutaraldehyde in 0.05 M PB, pH 7.2, at RT).

Brains were extracted, blocked and post-fixed for 4 h. The blocked brains were stored overnight in 10% sucrose in 0.05 M PB for cryoprotection at 4°C. Next, the brains were gelatin-embedded (10% gelatine, 10% sucrose in distilled water), hardened in 10% formaldehyde with 30% sucrose for 3 h (RT) and stored until sectioning in 30% sucrose in 0.05 M PB at 4°C.

With a freezing microtome, 40 μm transverse sections were cut and serially collected in 0.1 M PB, pH 7.2 in eight glass vials. Free floating sections were processed for CTb immunohistochemistry as follows. First, sections were thoroughly rinsed in TBS+ (0.05 M Tris, 0.5 M NaCl, 0.5% Triton X-100, pH 8.6), and were subsequently incubated for 48 h in the dark at 4°C under constant agitation with anti-CTb (1:15,000 in TBS+, List Lab.). Hereafter, sections were thoroughly rinsed with TBS+, and incubated with biotinylated donkey-anti-goat antibodies (List Biological Lab.; 1: 2000 in TBS+) for 2 h at RT. Then, rinsed sections were incubated with the avidine-biotine complex (ABC Elite Kit, 1:100 in TBS+, Vector Lab., Burlingame, CA) for 2 h (RT). Following a subsequent rinses in 0.05 M Tris-HCl, pH 7.3, sections were reacted with diaminobenzidine (37.5 mg DAB per 150 ml Tris-HCl with 25 μ l 30% H_2_O_2_) for 20–30 min. This reaction was stopped by rinsing the sections in 0.1 M PB (pH 7.2). Finally, the gold-lectin labeling was intensified by a silver-enhancement procedure (Aurion, Wageningen, Netherlands: Ruigrok and Apps, 2007). Sections within a vial were mounted serially, air dried, counterstained with thionine and coverslipped with Permount™ (Fisher Scientific).

### Analysis of sections

**Figure 1:**
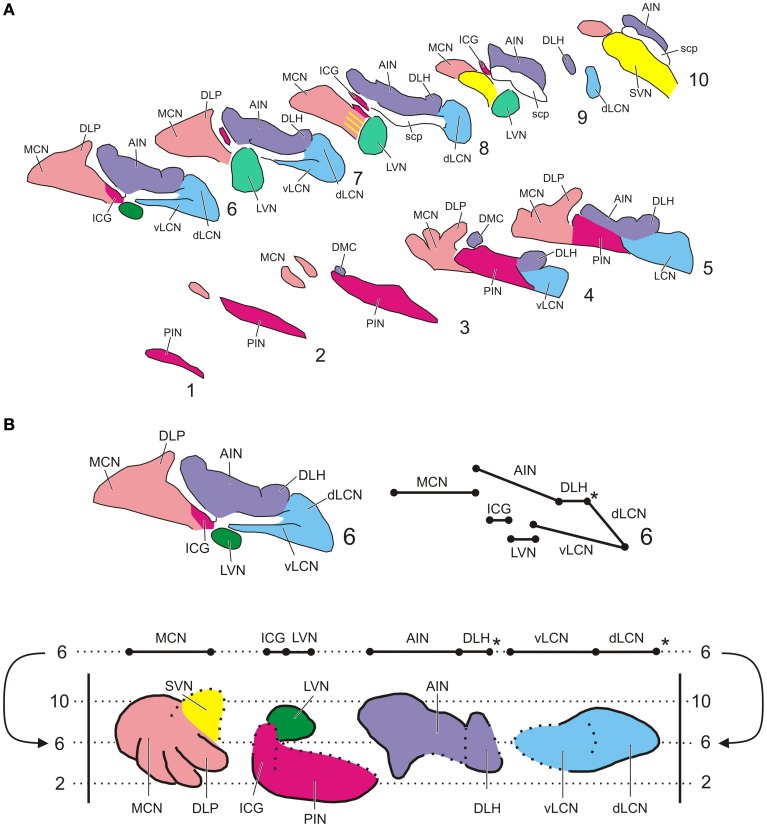
**Standardized diagrams of the right hand cerebellar nuclear complex of the rat. (A)** Series of transverse sections from caudal (1) to rostral (10) separated by 160 micron (adapted after Ruigrok and Voogd, 2000). Medial is to the left. **(B)** Construction of the standardized diagram of a dorsal view of the separated cerebellar nuclei (adapted after Ruigrok and Voogd, 2000). Upper diagrams show how level 6 of the transverse diagrams is transposed into the bottom diagram. Note that the dorsal and ventral parts of the LCN are unfolded. ^*^Denotes a break in the cellular chain from dorsal LCN to the lateral aspect of the DLH. Abbreviations: AIN, anterior interposed nucleus; dLCN, dorsolateral part of LCN; DLH, dorsolateral hump; DLP, dorsolateral protuberance; DMC, dorsomedial crest; ICG, interstitial cell groups; LCN, lateral cerebellar nucleus; LVN, lateral vestibular nucleus; MCN, medial cerebellar nucleus; PIN, posterior interposed nucleus; scp, superior cerebellar nucleus; SVN, superior vestibular nucleus; vLCN, ventromedial part of LCN.

Sections were analyzed with a motorized Olympus BH-2 light microscope equipped with a Lucivid™ miniature camera and a plotting system using Neurolucida™ software (MicroBrightfield, Inc., Colchester, VT). Using this system the resulting CN labeling contralateral to the injection site was plotted indicating every labeled neuron in the CN in a one out of eight series of sections. In this way five representative sections throughout the rostrocaudal length of the CN and separated by 320 micron were analyzed using a 40× objective. The resulting plots were subsequently compiled into a standardized diagram of the flattened, stretched-out CN as based on transverse sections (Figure 1). The lateral vestibular nucleus (LVN) was not included in the analysis. Injection sites were indicated on standardized diagrams.

In order to obtain an estimate of the degree of collateralization, the percentage of double-labeled cells was calculated based on the number of labeled cells in an overlapping area. To do this, a contour enveloping the area in which both types of labeled neurons were found was made. Labeled neurons were defined as positioned within an overlapping areas if they were separated by less than 300 micron. the surface of the areas indicating labeling of either type in the flattened CN diagrams was determined as well as the surface of the overlapping area. Subsequently, the number of labeled cells of either type (single or double labeled) was determined (Table 1).

**Figure 2:**
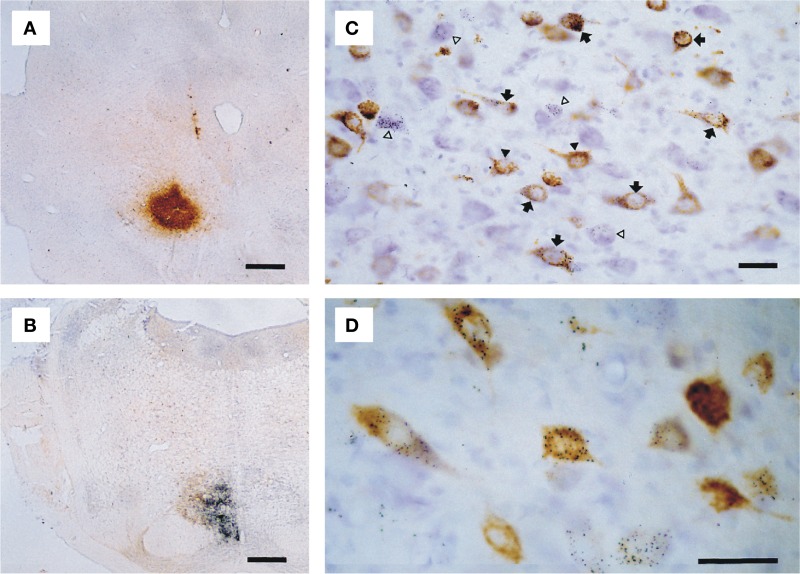
**Microphotographs showing examples of double labeling experiment T64. (A)** Iontophoretic injection with CTb centered on the RN. **(B)** Pressure injection with gold-lectin injection centered on the ipsilateral NRTP. **(C)** Detail of resultant labeling in the contralateral lateral cerebellar nucleus; neurons only labeled with gold-lectin are indicated by open arrow heads, neurons only labeled with CTb are indicated by black arrow heads, arrows depict neurons that contain both labels. **(D)** Higher magnification of single and double-labeled cells within the anterior interposed nucleus. Scale bars equal 500 μm in **(A,B)** and 25 μm in **(C,D)**.

Selected sections were photographed with a Leica DMR microscope equipped with a digital camera (Leica DC300) and the photopanel (Figure 2) was constructed with CorelDraw™ 11.0 after some correction for brightness and contrast in CorelPhotopaint™ 11.0.

### Nomenclature and definition of areas

Identification and nomenclature of the subdivisions of the IO and of the CN (Figure 1) was based on Ruigrok (2004) and Voogd (2004), respectively. Outlines of the NRTP were based on Mihailoff et al. (1981). The delineation of the RN and adjacent prerubral area were based on descriptions by Reid et al. (1975) and Ruigrok (2004).

## Results

### General aspects of injection sites and resulting labeling

The iontophoretic CTb injection sites appeared as globular shaped concentrations of a brownish, fine granular substance, with centrally the darkest coloring and the highest concentration of granules, gradually fading toward the edges but with well outlined borders (Figure 2A). Gold-lectin injections consisted of an irregularly shaped, but usually well-outlined aggregate of small black particles (Figure 2B).

All injections resulted in labeling of somatic neuronal contours in the CN. CTb labeled neurons were readily identified in thionine counterstained sections by their content of brown granules in the somata and proximal dendrites (Figures 2C,D), which frequently gave the soma a solidly brown appearance. Silver-intensified gold-lectin containing neurons were readily identified and discriminated from unlabeled or CTb labeled neurons by their content of small, intensely black particles in the cytoplasm (Figures 2C,D). Gold-particles were evenly distributed through the somata and proximal-most parts of dendrites. Double-labeled neurons were identified by the simultaneous occurrence of coarse brown granules and fine black particles in the same neuronal contour (Figures 2C,D). No indications were found that the resultant retrograde labeling was biased by the choice of tracer (Koekkoek and Ruigrok, 1995; Ruigrok et al., 1995b; Ruigrok and Apps, 2007).

### Collateralization of nucleo-bulbar projections

All injections resulted in labeled neurons that were distributed throughout several of the main CN. Indeed, it would appear to be a rule that projections from a particular CN subnucleus are distributed to at least several of the investigated areas. This would imply that individual neurons located within such an area could collateralize to all these regions, or, when collateralization is absent, that every type of projection stems from a distinct, but intermingled, population of cells within such a CN subnucleus. Details of the distribution of single and double labeled neurons resulting from the six different combinations of injections will be presented below (also see Table 1).

#### Combinations involving the inferior olive

**Figure 3:**
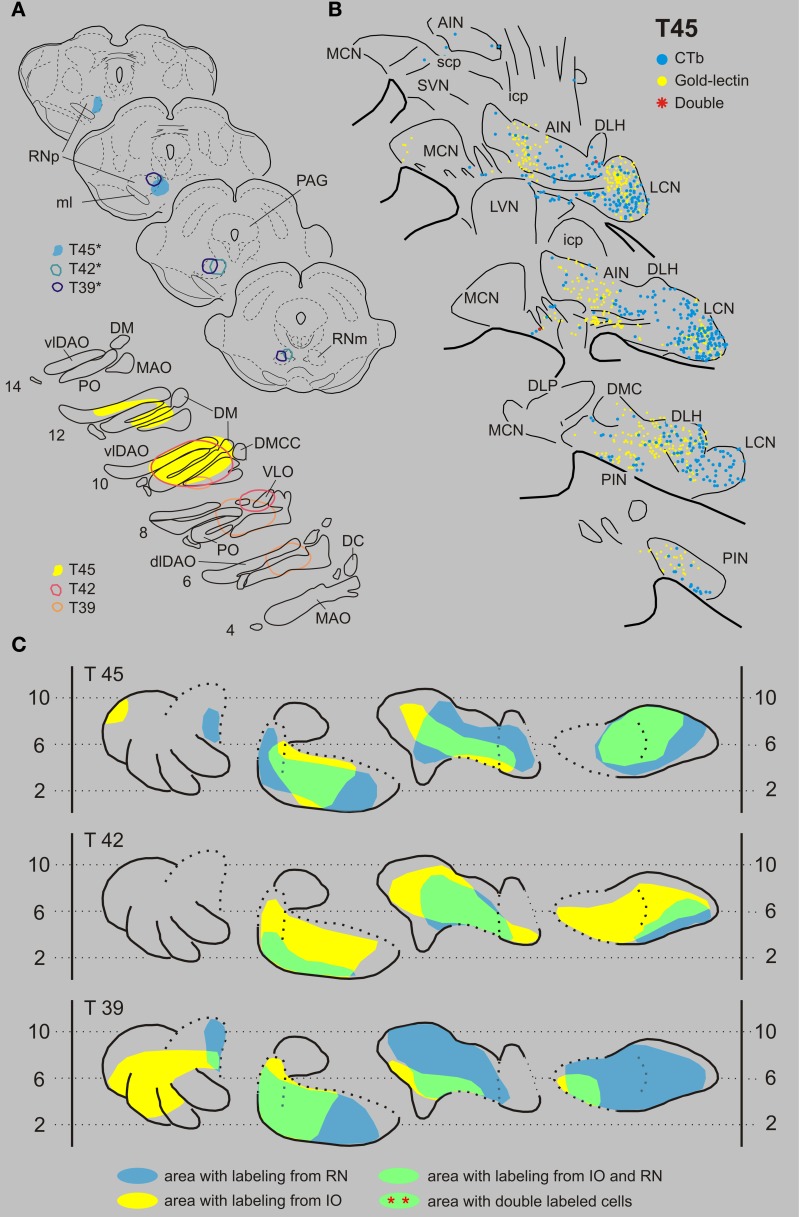
**Diagrams depicting results of the combination IO and RN. (A)** Injection sites of all three analyzed cases. Upper left hand panels show the injection sites within transverse diagrams of the midbrain. Bottom panels show the related injection sites as placed in the left inferior olivary complex, depicted in standardized diagrams (adapted after Ruigrok and Voogd, 2000). In this combination all CTb injection sites (case number indicated with^*^) were made in the RN, while gold-lectin was injected in the IO. **(B)** Resultant plots of 5 equidistant (320 micron) sections (from caudal, bottom, to rostral, top) showing labeled neurons within the contralateral CN of case T45. Every symbol indicates a single labeled neuron. **(C)** Single and double-labeling of case T45 as well as from the other two cases (T42 and T39) indicated in standardized diagrams shown in Figure 1B. Areas containing retrograde labeling from the RN are indicated in blue, whereas labeling from the IO are indicated in yellow. Areas containing cells with either label are indicated in green. However, in all cases virtually no double labeled neurons were encountered (Table 1). Abbreviations: AIN, anterior interposed nucleus; DC, dorsal cap; dfDAO, dorsal fold of dorsal accessory olive; dLCN, dorsolateral part of LCN; DLH, dorsolateral hump; DLP, dorsolateral protuberance; DM, dorsomedial group; DMC, dorsomedial crest; DMCC, dorsomedial cell column; ICG, interstitial cell groups; icp, inferior cerebellar peduncle; LCN, lateral cerebellar nucleus; LVN, lateral vestibular nucleus; MAO, medial accessory olive; MCN, medial cerebellar nucleus; ml, medial lemniscus; PAG, periaqueductal gray; PIN, posterior interposed nucleus; PO, principal olive; RN, red nucleus; RNm, magnocellular red nucleus; RNp, parvicellular red nucleus; scp, superior cerebellar nucleus; SVN, superior vestibular nucleus; vLCN, ventromedial part of LCN; vlDAO, ventral fold of dorsal accessory olive; VLO, ventrolateral outgrowth.

***Inferior olive and red nucleus***. Figures 3A,B shows the injection sites and resultant retrograde labeling in the contralateral CN of case T45. The olivary injection with gold-lectin covered large areas of the principal olive (PO), central part of the medial accessory olive (MAO) and dorsal accessory olive (DAO) and resulted in concomitant labeling within large areas of the lateral cerebellar nucleus (LCN), posterior interposed nucleus (PIN), and anterior interposed nucleus (AIN). Only a few labeled cells were observed in the rostromedial part of the MCN. The CTb injection was centered on the medial aspect of the rostral halve of the RN. Retrogradely labeled cells were mostly confined to the LCN, sparing its ventromedial-most part. More sparsely distributed neurons were located within both interposed nuclei. Large areas of overlap are noted, especially when the labeled areas are indicated in the flattened and stretched-out diagrams of the CN (Figure 3C). Within overlapping areas, gold-lectin labeled neurons were completely intermingled with the CTb labeled neurons (also see Teune et al., 1995). However, out of a total of 990 labeled neurons only two double-labeled cells were found (Table 1). Obviously, not all retrogradely labeled cells were located in regions were both types of cells are encountered (i.e., in an “overlapping” area). Therefore, in order to obtain a better estimate of the chance that a CN neuron can collateralize to both injected areas, the percentage of double-labeled cells was calculated based on the number of labeled cells in an overlapping area (see Materials and Methods). Thus, for case T45, the two double-labeled neurons were located in an overlapping area where 501 gold-lectin-labeled neurons and 340 CTb-labeled neurons were found in the analyzed sections. This would indicate that in the overlapping areas (i.e., central PIN, lateral AIN, and rostral LCN) 0.40% of the gold-lectin-labeled neurons were double-labeled and 0.59% of the neurons that were labeled from the RN injection (Table 1).

In two additional cases (T39 and T42, for injection sites see Figure 3A) overlapping areas were also found in PIN, AIN and in a relatively small area of the LCN (Figure 3C). Similar to case T45 virtually no double-labeled cells were encountered (0 out of a total of 1121 and 1 out of 870 labeled neurons in T 42 and T 39, respectively, see also Table 1).

**Figure 4:**
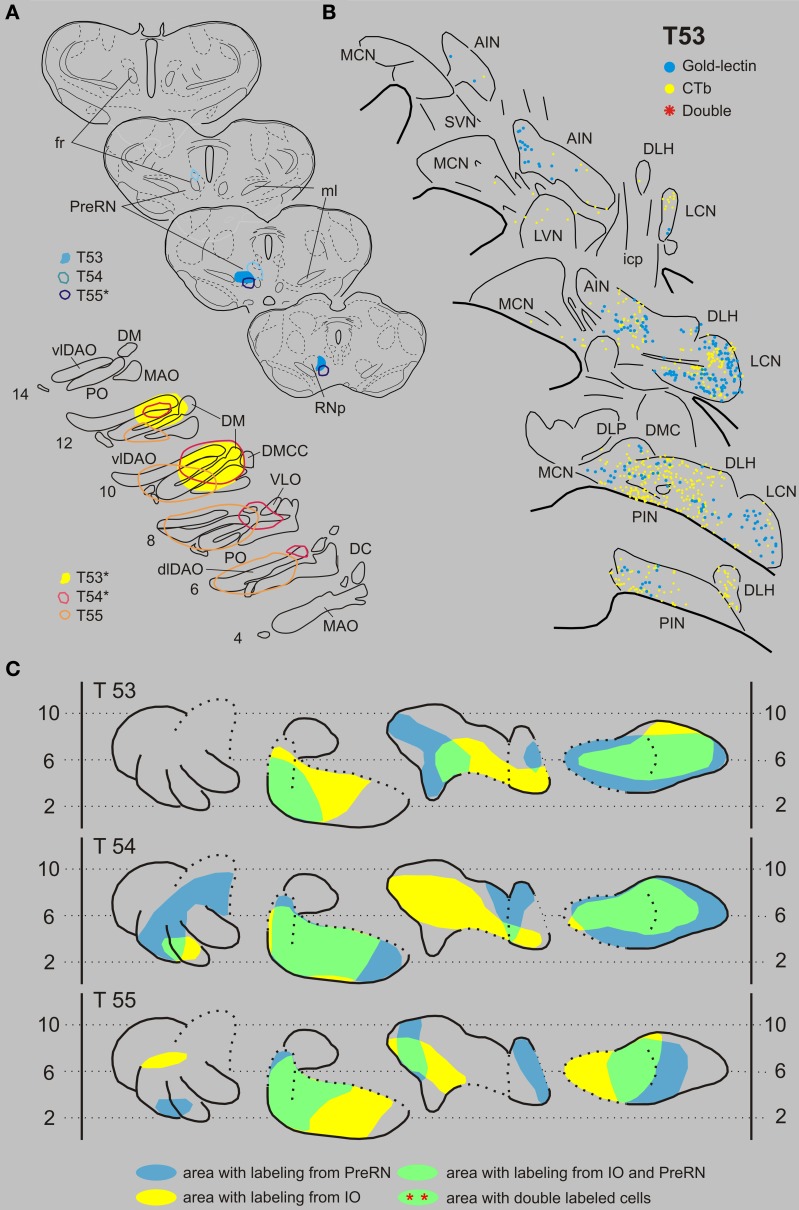
**Diagrams depicting results of the combination IO and the PreRN. (A)** Injection sites of all three analyzed cases. Upper left hand panels show the injection sites within transverse diagrams of the mesodiencephalon. Bottom panels show the related injections sites in the IO. CTb injections (case number indicated with^*^) were made in the IO in cases T53 and T54, but in the PreRN in case T55. **(B)** Plots of 5 equidistant (320 micron) sections showing labeled neurons within the contralateral CN of case T53. **(C)** Overview of the distribution of retrogradely labeled neurons of case T53 and of the two additional cases (T54 and T55) in standardized diagrams of the CN. Areas containing labeled neurons from the PreRN and IO are indicated in blue and yellow, respectively. In all cases virtually no double-labeled neurons were encountered (Table 1). Abbreviations: PreRN, prerubral area; fr, fasciculus retroflexus; SN, substantia nigra. Conventions and other abbreviations as in Figure 3.

***Inferior olive and prerubral area***. Case T53 was chosen as a typical example of an IO-PreRN injection combination (Figures 4A,B). Here, gold-lectin was injected in the prerubral region but also involved the medial part of the parvicellular part of the RN (RNp). CTb had been injected in the central and medial aspects of the inferior olivary complex. Although within the overlapping regions of the CN, i.e., the medial part of the PIN, central part of the AIN and most of the LCN, 336 CTb-labeled and 217 gold-lectin-labeled neurons were counted in the analyzed sections, no double-labeled cells were observed (Table 1). A similar picture emerged in two additional cases (Figures 4A,B: T54 and T55). Again, despite extensive CN areas where both cell types were intermingled not a single (T54) and only one (T55) double-labeled neuron was found (Table 1, Figure 9).

**Figure 5:**
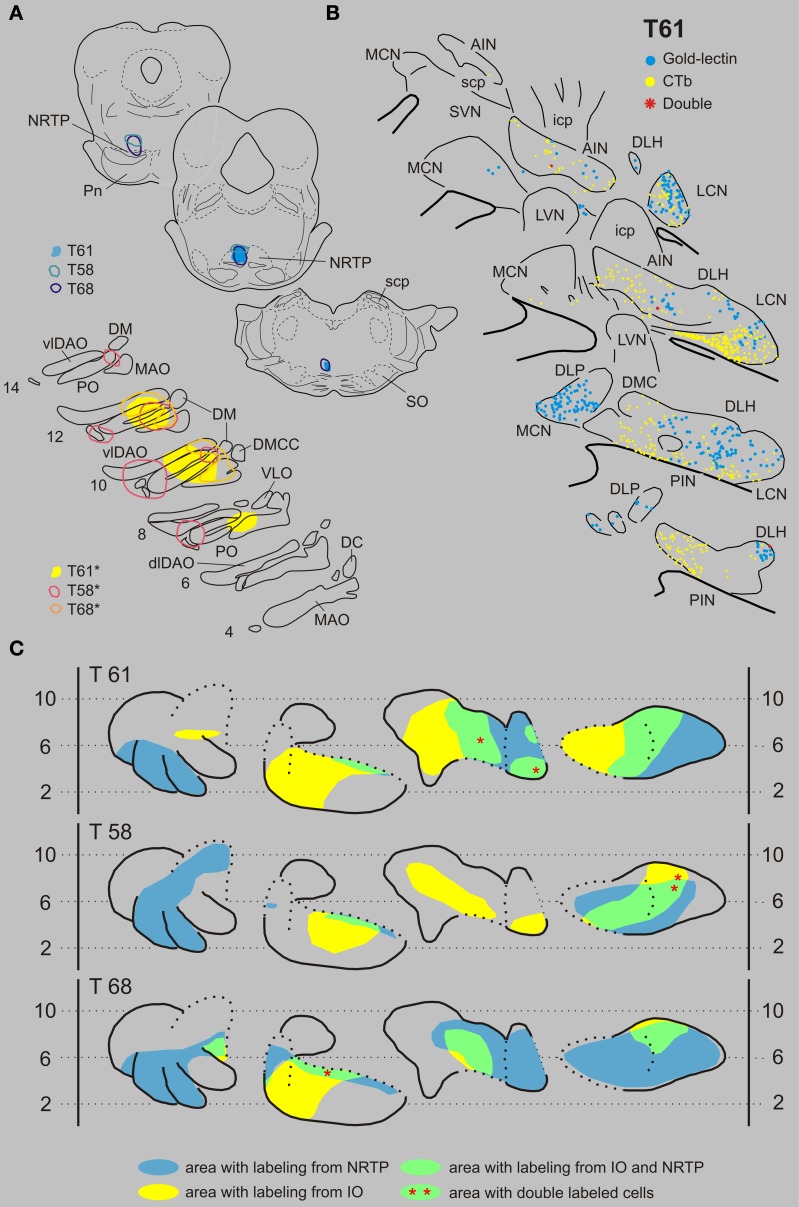
**Diagrams depicting results of the combination IO and the NRTP. (A)** Injection sites of all three analyzed cases. Upper left hand panels show the injection sites within transverse diagrams of the pontine region. Bottom panels show the related injections sites in the IO. CTb injections (case number indicated with^*^) were made in the IO of all three cases. **(B)** Plots of 5 equidistant (320 micron) sections showing labeled neurons within the contralateral CN of case T61. **(C)** Overview of the distribution of retrogradely labeled neurons of case T61 and of the two additional cases (T58 and T68) in standardized diagrams of the CN. Areas containing labeled neurons from the NRTP and IO are indicated in blue and yellow, respectively. Only a few double-labeled neurons were encountered (Table 1). Abbreviations: NRTP, nucleus reticularis tegmenti pontis; Pn, basilar pontine nuclei; SO, superior olivary complex. Conventions and other abbreviations as in Figure 3.

***Inferior olive and nucleus reticularis tegmenti pontis***. Three examples of cases with injections in the IO and NRTP are shown in Figure 5. Case T61 received a gold-lectin injection in the medial half of the NRTP and two iontophoretic CTb injections were centered on the central MAO and DAO, respectively, but also involved parts of the PO. CN regions that contained both types of retrogradely labeled neurons were located in the lateral part of the AIN and within the ventrolateral part of the LCN. These overlapping regions contained 187 gold-lectin and 196 CTb labeled neurons. Three of these neurons, which were all small-sized, contained both labels and were located in the caudal half of the dorsolateral hump (DLH: 2 cells) and lateral AIN (1 cell). In case T58 (CTb injections in IO centered on lateral bend in PO and involving adjacent DAO and an additional injection centered on central MAO, and gold-lectin centered on the rostroventral aspect of the medial NRTP and involving the dorsomedial part of the basilar pontine nuclei, Figure 5A), the overlapping area was mainly restricted to the LCN where 2 out of 99 gold-lectin and 70 CTb-labeled neurons were double-labeled. The CN of case T68 harbored only two double labeled neurons (out of a total plotted number of 680 labeled neurons), which were both located in the rostroventral aspect of the PIN, directly bordering the roof of the 4th ventricle (Figures 5, 9 and Table 1).

#### Remaining combinations that involve the nucleus reticularis tegmenti pontis

**Figure 6:**
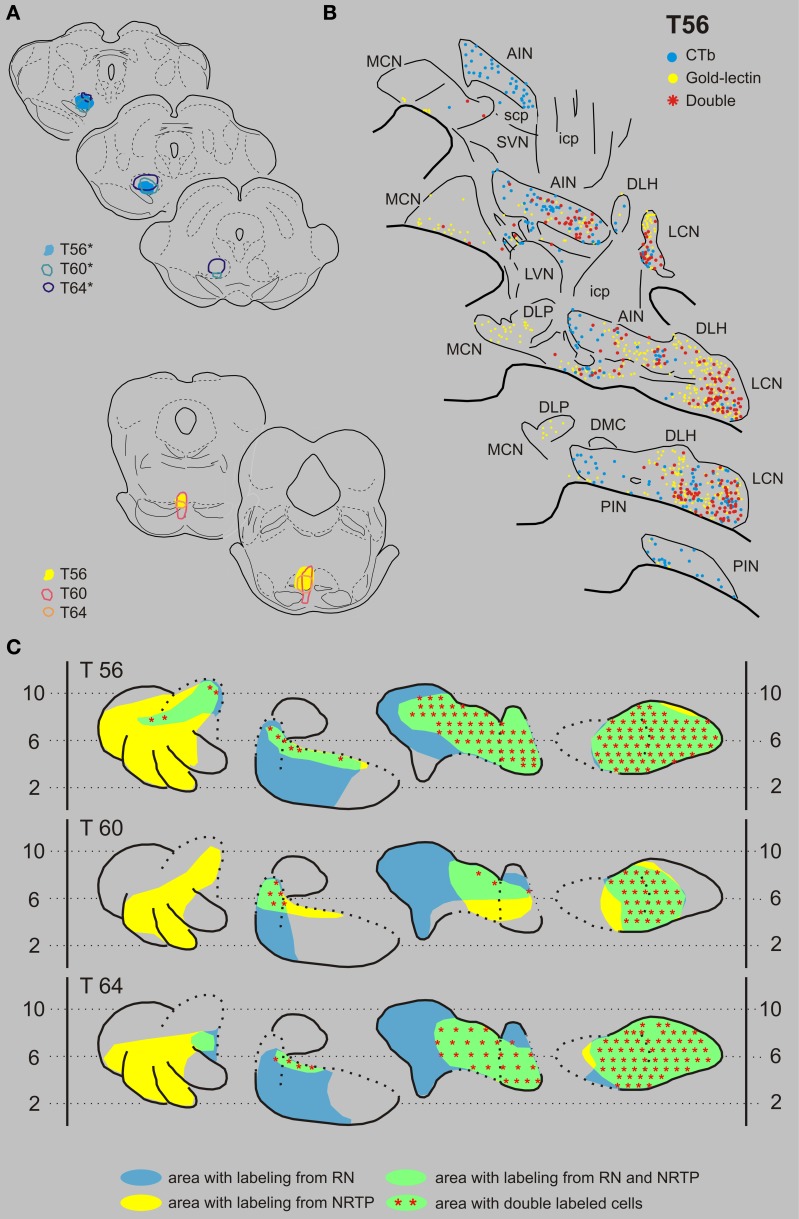
**Diagrams depicting results of the combination NRTP and the RN. (A)** Injection sites of all three analyzed cases. Upper left hand panels show the injection sites within transverse diagrams of the midbrain. Bottom panels show the related injections sites in the NRTP. CTb injections (case number indicated with^*^) were made in the RN of all three cases. **(B)** Plots of 5 equidistant (320 micron) sections showing labeled neurons within the contralateral CN of case T56. **(C)** Overview of the distribution of retrogradely labeled neurons of case T56 and of the two additional cases (T60 and T64) in standardized diagrams of the CN. Areas containing labeled neurons from the RN and NRTP are indicated in blue and yellow, respectively. Note that all overlapping areas (green) contain double labeled neurons (red symbols). Conventions and abbreviations as in Figures 3–5.

***Nucleus reticularis tegmenti pontis and the red nucleus***. Figures 6A,B displays the results of case T56 with a CTb injection that was centered on the RNp but also incorporated part of the magnocellular division of the RN (RNm), and a gold-lectin injection in the NRTP that only just encroached upon the dorsal part of the basilar pontine nuclei. CN regions where both types of retrogradely labeled cells were found encompassed most of the LCN and AIN, a small strip of cells in the rostroventral PIN and to some degree within the rostralateral part of the MCN. Double-labeled neurons were present in all areas of the CN where the two populations overlapped. Of 407 CTb-labeled cells that were located in these areas, 225 (55%) were also labeled with gold-lectin. The plotted number of cells projecting to the NRTP (gold-lectin labeled) in these overlapping areas was 568, implying that approximately 40% of these neurons collateralize to the RN. Two additional cases, T60 and T64, had CTb injections that centered on most of the RNm and RNp and gold-lectin injections into the medial NRTP (Figures 2A,D, 6A,C). As in T56, many double-labeled neurons were encountered in the LCN, lateral half of the AIN, and in the rostral part of the interstitial cell groups (ICG) and rostroventral part of the PIN (also see Figure 9 and Table 1).

**Figure 7:**
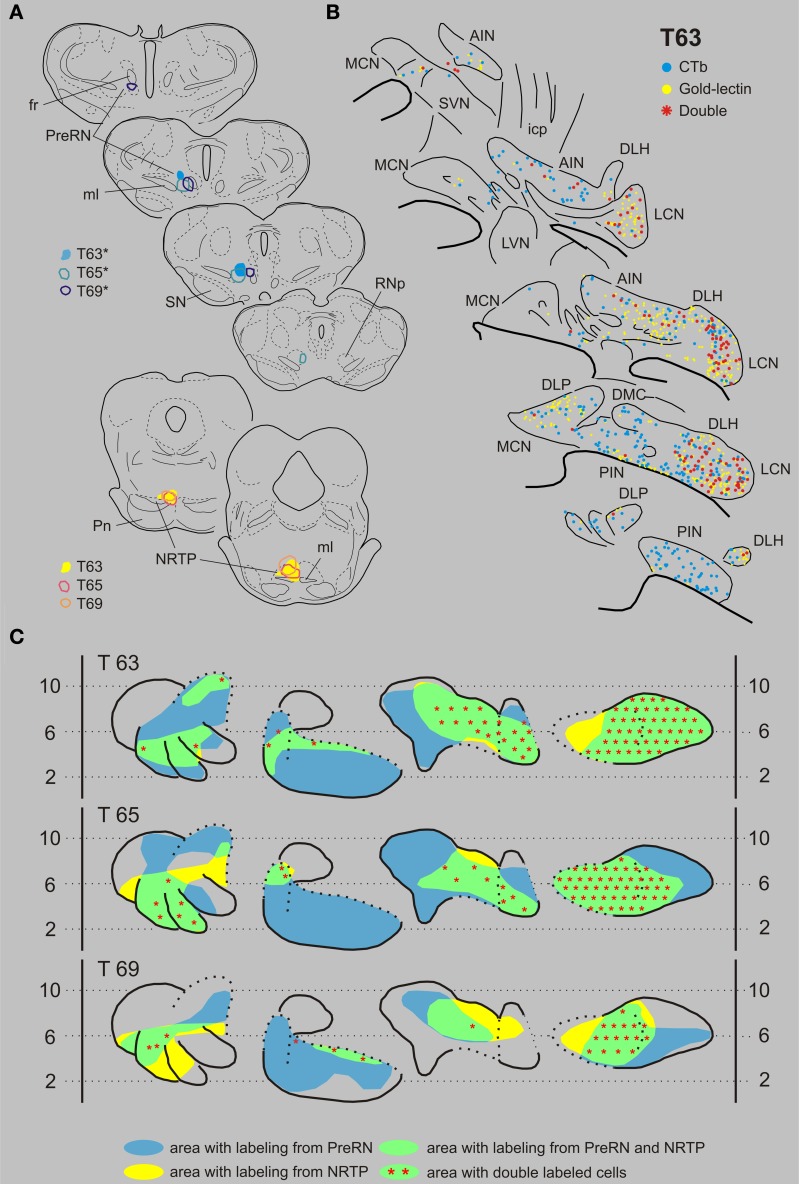
**Diagrams depicting results of the combination NRTP and the PreRN. (A)** Injection sites of all three analyzed cases. Upper left hand panels show the injection sites within transverse diagrams of the mesodiencephalon. Bottom panels show the related injections sites in the NRTP. CTb injections (case number indicated with^*^) were made in the PreRN of all three cases. **(B)** Plots of 5 equidistant (320 micron) sections showing labeled neurons within the contralateral CN of case T63. **(C)** Overview of the distribution of retrogradely labeled neurons of case T63 and of the two additional cases (T65 and T69) in standardized diagrams of the CN. Areas containing labeled neurons from the PreRN and NRTP are indicated in blue and yellow, respectively. Note that all overlapping areas (green) contain double labeled neurons (red symbols). Conventions and abbreviations as in Figures 3–5.

***Nucleus reticularis tegmenti pontis and the prerubral area***. T63 serves as a typical example of a case with a combination of injections in the NRTP (with gold-lectin) and in the prerubral area (Figures 7A,B). Gold-lectin labeling in the CN, as in the other cases where the NRTP was injected, was found in many areas but was essentially absent in the dorsomedial MCN and within the PIN. CTb-labeling was prominent within the LCN and PIN, whereas more scattered cells were encountered in the AIN/DLH and the caudal MCN. Thus, most of the LCN, the lateral AIN/DLH, caudal MCN and the rostroventral PIN/rostral ICG constitute regions where both cell types were intermingled. Indeed, in all these areas double-labeled cells were encountered, contributing to about 25% of the number of gold-lectin labeled cells (projecting to the NRTP) and to 30% of the CTb labeled cells (Table 1, Figure 9). Basically similar results were obtained in two additional cases (cases T65 and T69, see Figures 7A,C). Note that in all cases the number of double-labeled cells appears highest in the LCN.

#### Combination of red nucleus and prerubral area

**Figure 8:**
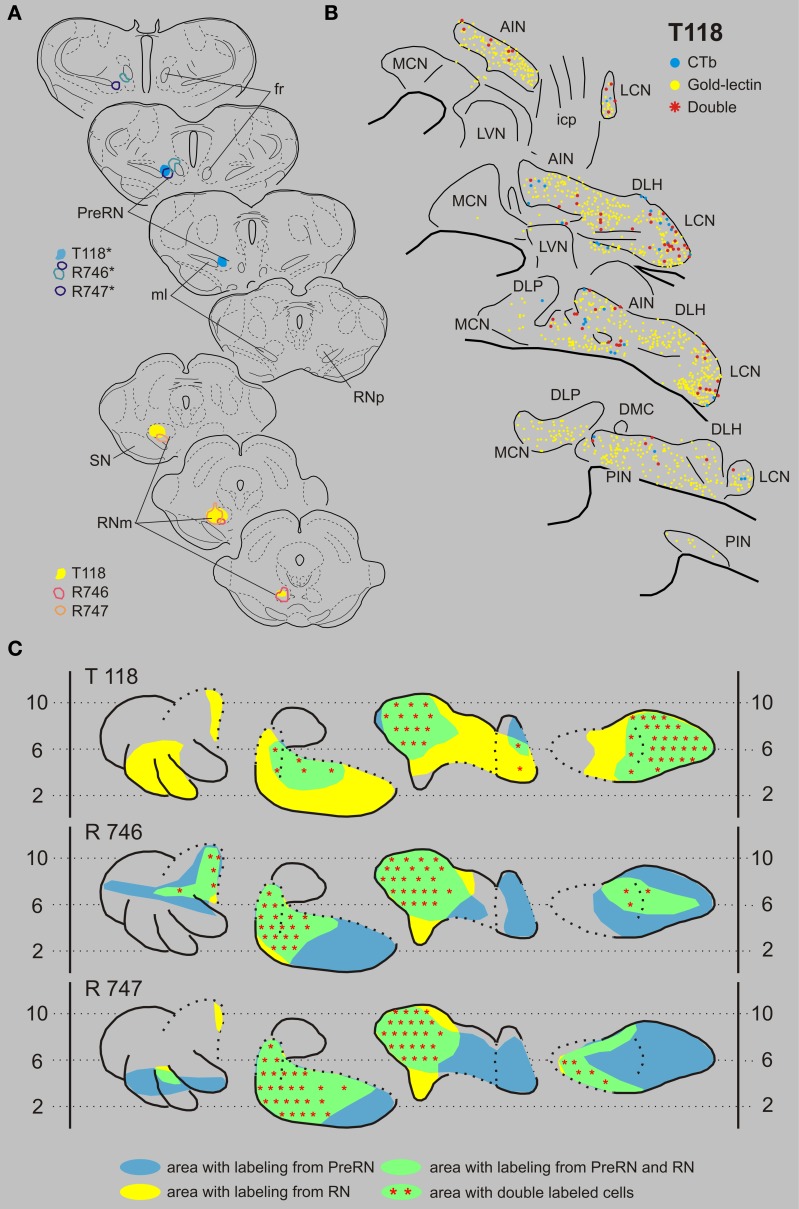
**Diagrams depicting results of the combination RN and the PreRN. (A)** Injection sites of all three analyzed cases. Upper left hand panels show the injection sites within transverse diagrams of the mesodiencephalon. Bottom panels show the related injections sites in the RN. CTb injections (case number indicated with^*^) were made in the PreRN of all three cases. **(B)** Plots of 5 equidistant (320 micron) sections showing labeled neurons within the contralateral CN of case T118. **(C)** Overview of the distribution of retrogradely labeled neurons of case T118 and of the two additional cases (R746 and R747) in standardized diagrams of the CN. Areas containing labeled neurons from the RN and PreRN are indicated in blue and yellow, respectively. Note that all overlapping areas (green) contain double labeled neurons (red symbols). Conventions and abbreviations as in Figures 3–5.

**Figure 9:**
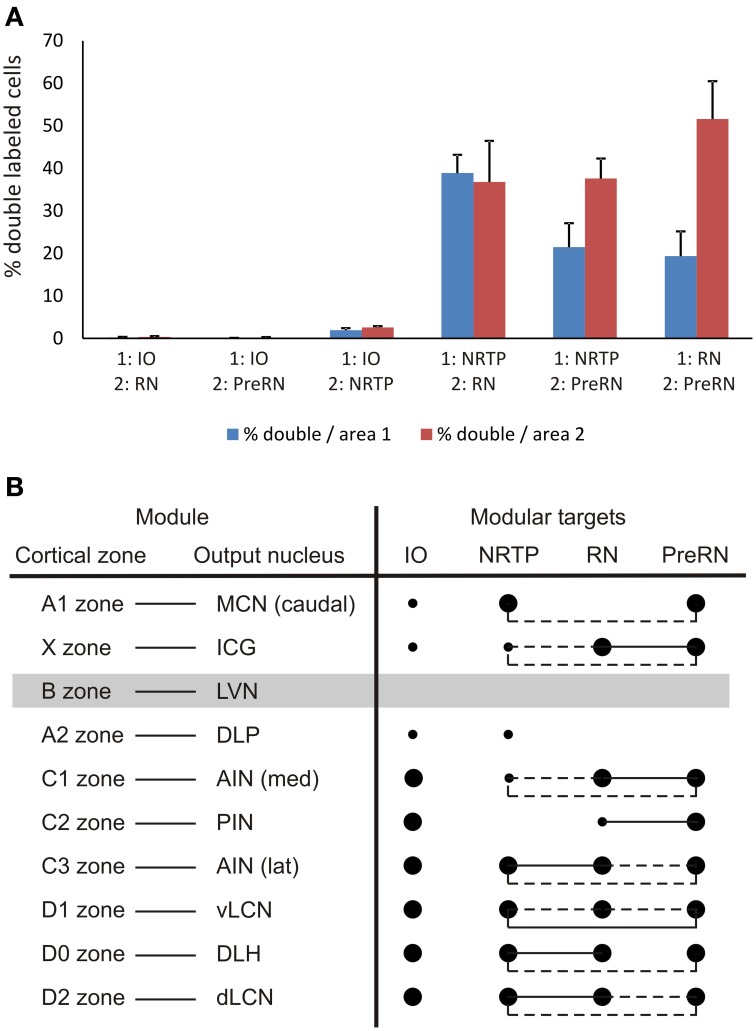
**(A)** Diagram depicting the average percentage (+SEM) of double-labeled neurons found in overlapping areas for all six combinations, based on the data of Table 1. Blue bars refer to the percentage of double labeled cells that project to the first mentioned area of the injection combination but containing both labels. Red bars refer to the percentage of double-labeled cells that project to the other injection site. **(B)** Overview of projection and collateralization patterns from different parts of the CN to the four investigated brainstem areas and related to the modular organization of the rat cerebellum. Filled large and small circles indicate major and minor projections, respectively, as noted between the listed CN areas and the investigated brainstem areas. Circles linked by pulled or hatched lines indicate that many or some double-labeled neurons, respectively, were encountered in the part of the CN of the same row. The lateral vestibular nucleus (LVN), output nucleus of the B zone, was not specifically studied in the present study (gray bar).

In this combination gold-lectin injections were made that were centered on the RN while the CTb injections were centered on the PreRN. Care was taken that in these selected cases the injection sites were not overlapping. The three selected cases are presented in Figure 8. The PreRN injection of case T118 did not incorporate the confines of the RNp (Figure 8A) and resulted in many CTb-labeled neurons in the rostrodorsal LCN, medial AIN, and, more sparsely, in the rostral PIN. Neurons labeled from a rather large gold-lectin injection into the RN were observed throughout most of the cerebellar nuclear complex except the rostral MCN and ventromedial LCN (Figures 8B,C). In all overlapping areas, i.e., dorsal LCN, medial AIN en rostral PIN, double-labeled neurons were encountered, amounting to about 10% of the gold-labeled cells but to almost 70% of the CTb labeled neurons (Figures 8, 9, Table 1). In two additional cases, the PreRN injections were centered just between the medial lemniscus and the retroflex bundle in case R746, and slightly more dorsomedially in case R747 (Figure 8A). The gold-lectin injections were both centered on the caudal aspect of the RNm. In both cases overlapping areas and many double-labeled neurons were observed in the medial halves of the AIN and PIN/ICG and, more sparsely, in the LCN (Figures 8, 9, Table 1).

## Discussion

### Objective of this study

This study was prompted by mounting evidence that the basis for cerebellar functioning may be found in its modular organization (Voogd and Bigaré, 1980; Apps and Garwicz, 2005; Apps and Hawkes, 2009; Ruigrok, 2011). As such, the distribution of the output of these modules by way of the cerebellar or vestibular target nuclei became a point of interest (Teune et al., 2000). In addition, it is evident that further identification of the level of collateralization of individual neurons would be necessary in order to evaluate the impact of cerebellar output. Here, we have selected four areas in the brainstem, which are known to receive cerebellar input but, as based on their own output characteristics, are very different in their function. The RN was chosen as a premotor nucleus since most of its neurons will enter the rubrospinal (or rubrobulbar) tract (Ruigrok, 2013) and because it is a compact and well delineated area. Other CN target areas with a premotor function that could have been selected would be e.g., the “motor” thalamus, the medial reticular formation, or the superior colliculus (Ruigrok, 2013). Many of the remaining projections appear to enter either directly or indirectly the cerebellar circuitry. Cerebellar projections to the NRTP but also to the basilar pontine nuclei (Mihailoff et al., 1989; Ruigrok, 2004), will influence cerebellar mossy fiber activity, whereas the source of climbing fibers, the IO, also receives a direct and prominent cerebellar nuclear projection. However, the IO is also heavily afferented by areas located at the mesodiencephalic junction such as the nucleus of Darkschewitsch, the prerubral area, the accessory oculomotor nucleus and the interstitial nucleus of the medial longitudinal fascicle (for review see Ruigrok, 2004). These areas are known to receive cerebellar input (Berretta et al., 1993; De Zeeuw and Ruigrok, 1994; Ruigrok and Voogd, 1995). Indeed, stimulation of the brachium conjunctivum in cat can result in disynaptic activation of olivary cells (Ruigrok and Voogd, 1995; Bazzigaluppi et al., 2012). Hence, as an excitatory preolivary area, the prerubral area was included in our study on the degree of convergence and divergence of cerebellar nuclear projections.

The results show that cerebellar nuclear neurons collateralize extensively to the NRTP, innervated by the crossed descending branch of the scp, and to the RN and prerubral area (PreRN), both provided by its crossed ascending branch. Moreover, a large proportion of the CN neurons projecting to RN also have terminations in the prerubral area. Neurons that provide an input to the IO, however, do not collateralize to these areas.

### General aspects of CN projections to IO, NRTP, RN, and prerubral area

#### Inferior olive

Nucleo-olivary neurons in general can be distinguished from other cerebellar nuclear relay neurons by their size and immunoreactive characteristics. They are small-sized, spindle shaped neurons and contain GABA as their main neurotransmitter (Chan-Palay, 1977; De Zeeuw et al., 1989; Fredette and Mugnaini, 1991). Since virtually all injections were made in the center of the IO complex (in order to maximize the amount of retrograde labeled cells without risk of tracer spillage to extraolivary regions), not much information is available on the results of injections into the caudal- and rostral-most aspects of the olivary complex. Nevertheless, the pattern of retrograde labeling following these injections was completely compatible with a detailed report by Ruigrok and Voogd (1990), which was based on anterograde tracing studies. In brief, injection sites involving the PO resulted in labeling in the LCN. Labeled neurons in the PIN were found after an injection involving the rostral MAO; the AIN contained labeled cells when the injection incorporated the DAO (excluding its dorsal fold). Injections involving the dorsomedial group resulted in labeling in the DLH. The organization of the nucleo-olivary projections appears to be neatly reciprocated by a matching olivonuclear projection by the climbing fiber collaterals (Ruigrok, 1997; Ruigrok and Voogd, 2000; Sugihara and Shinoda, 2007).

#### Nucleus reticularis tegmenti pontis

In all cases, most of the retrogradely labeled neurons were observed in the LCN. In agreement with an anterograde tracing study by Angaut et al. (1985) we noted that injections that were centered on the caudal part of the NRTP tended to result in labeling of neurons in the rostrodorsal part of the LCN, whereas labeling in caudomedial LCN resulted from more rostrally placed injections. Within the interposed nuclei most labeling was observed within the DLH and lateral part of the AIN. Virtually no projections to the NRTP originate from the PIN as was already noted by Torigoe et al. (1986). In our material, however, there was some, but consistent, retrograde labeling of neurons located directly dorsal to the roof of the 4th ventricle, a transition area that is continuous with labeling in the rostral part of the ICG located between the medial part of the AIN and the MCN.

The projections from the MCN onto the NRTP have been described as relatively sparse and to terminate mainly in a dorsomedial column throughout the NRTP (Cicirata et al., 1982; Watt and Mihailoff, 1983). In our retrograde study we have noted that the NRTP injections, which all involved the medial part of the NRTP, resulted in labeled neurons that were usually confined to the caudal pole of the MCN, but sometimes included the base of the DLP and the rostrolateral MCN.

#### Red nucleus

The organization of cerebellar nuclear projections to the RN in the rat has been investigated using anterograde and retrograde tracing techniques (for review see Ruigrok, 2004). Basically, the results presented here are in agreement with earlier studies. The medial part of the AIN projects to the ventrolateral RNm, the lateral AIN projects to the dorsomedial RNm and the medial part of the PIN contains labeled neurons when the medial border of the RN was included in the injection site. Although our injections that were centered on the RNp never resulted in exclusive labeling of the LCN, we concur with other authors that most cerebellar terminals to this part of the RN originate from the LCN (Flumerfelt, 1978; Angaut and Cicirata, 1988). The MCN is usually devoid of labeling with exception of some neurons in the rostrolateral MCN. In some cases (e.g., T118, Figure 8) additional labeling within the MCN may have been caused by inadvertent spread of the tracer to overlying accessory oculomotor areas (Gonzalo-Ruiz and Leichnetz, 1987).

When the injection extended into the area directly lateral to the RNm (pararubral area: Paxinos and Watson, 2005), retrograde labeling involved a small area in the caudal part of the ventromedial LCN (Ruigrok and Cella, 1995; Teune et al., 2000). It is not known to what extent this pararubral region should be considered as a premotor or a preolivary region (Newman, 1985).

#### Prerubral area

The medial part of the mesodiencephalic junction has received attention as an area from which a prominent projection to the IO originates (for review see Ruigrok, 2004). This region includes the prerubral field (medial to the medial lemniscus and dorsal and lateral to the fasciculus retroflexus), the medial accessory oculomotor nucleus, the rostral part of the nucleus of Darkschewitsch, the subparafascicular nucleus and the rostral interstitial nucleus of the medial longitudinal fascicle. Here, this area is collectively referred to as prerubral area (PreRN). The rat PreRN, rather than the RNp, has been considered as a homolog of the primate parvicellular RN (Ruigrok, 2004; Onodera and Hicks, 2009).

Cerebellar projections to the mesodiencephalic junction have been mainly investigated in cat and appear to originate predominantly from the LCN and PIN, but projections to the nucleus of Darkschewitsch have also been shown to arise from AIN and MCN (Kawamura et al., 1982; Sugimoto et al., 1982; Onodera and Hicks, 1995; Teune et al., 2000). In accordance with these studies, injection with retrograde tracers in the PreRN mostly result in retrograde labeling in the LCN and PIN, but also involves the AIN including the DLH. LCN labeling included its ventromedial (parvicellular) part. In the PIN, labeling incorporated the ICG but also extended into the lateral and caudal pole of the nucleus. In some cases labeled cells were present in the rostrolateral MCN as well as in the caudal pole of this nucleus. We propose, as indeed demonstrated in the cat, that neurons in the PreRN mediate a major disynaptic excitatory pathway between the CN and the IO (De Zeeuw and Ruigrok, 1994; Ruigrok and Voogd, 1995).

### Collateralization of cerebellar nuclear output

Our experiments are in agreement with and extend the generally held concept that the CN harbor at least two different populations of projection neurons. One group, the nucleo-olivary neurons, consists of small-sized neurons which are distributed throughout the CN and reportedly all contain GABA as neurotransmitter (Angaut and Sotelo, 1989; De Zeeuw et al., 1989; Fredette and Mugnaini, 1991). The question remains, however, to what extent the nucleo-olivary neurons may collateralize to other cerebellar projection areas such as the bulbar reticular formation (Buisseret-Delmas et al., 1989), the thalamus or the basilar pontine nuclei and/or NRTP (Border et al., 1986; Aas and Brodal, 1990; Verveer et al., 1997).

The second group of projection neurons consists of medium- to large-sized cells which may show a rather extensive collateralization to these latter areas, while observing regional differences in their projection pattern. These neurons, apart from a group of glycinergic neurons in the rostral MCN (Bagnall et al., 2009), most likely, are all excitatory (Giuffrida et al., 1993; De Zeeuw and Ruigrok, 1994; Uusisaari and Knopfel, 2011). Below, we will discuss our observations in relation to these two main populations of cerebellar projection neurons.

#### Nucleo-olivary neurons

The collateralization of nucleo-olivary neurons to other brainstem regions has been investigated with both anatomical and electrophysiological techniques. The fluorescent double-labeling studies by Bentivoglio and Kuypers (1982), investigating cerebellar nuclear collateralization to the cervical cord, the caudal medulla, the mesencephalon (superior colliculus and RN) and the thalamus, were already suggestive of a non-collateralizing projection from small-sized neurons to the IO. However, definite conclusions were hampered by the large injection sites that covered sizable parts of the caudal medulla. In the cat, and in line with an unique nucleo-olivary projection, Legendre and Courville (1987) and Bharos et al. (1981) were unable to find nucleo-olivary neurons that project to the thalamus. Lee et al. (1989), on the other hand, found evidence of a small, collateral projection of nucleo-olivary neurons to the contralateral basilar pontine nuclei in the rat. This would be in line with observations in both the rat and the cat of a cerebellar derived GABAergic projection to the basilar pontine nuclei and including NRTP (Border et al., 1986; Aas and Brodal, 1990). Electrophysiologically obtained data in the rat (Berretta et al., 1991) appears to collaborate this notion but contrasts a similar study in the cat where only excitatory monosynaptic responses could be elicited in NRTP neurons after stimulation of the LCN (Kitai et al., 1976). Our own data indicate that only a very small portion of the nucleo-olivary fibers may collateralize to the NRTP. At best only 2 out of 70 labeled neurons in an overlapping region (case T 58) was double-labeled in a combination involving the NRTP and IO. It should be realized, however, that, although specifically for this reason gold-lectin was used as the injected tracer, the interpretation of a potential collateralization of nucleo-olivary fibers to the NRTP may be hampered by the risk of false positive double-labeling which may result from inadvertent uptake by damaged axons of the crossed descending limb of the scp that runs directly dorsal to and through the NRPT and which contains most of the nucleo-olivary fibers (Legendre and Courville, 1987; Ruigrok and Voogd, 1990). Indeed in the cat, an ultrastructural study failed to establish a GABAergic projection originating from the CN to the NRTP in the cat (Verveer et al., 1997). Nevertheless, especially since Lee et al. (1989) made use of a ventral approach to the BPN, thus minimizing the risk of false positive labeling, it would seem possible that a very small number of nucleo-olivary neurons truly may have collaterals to the basilar pontine nuclei and/or NRTP.

In the cat, several studies, mostly based on electrophysiological techniques have suggested that at least part of the nucleo-olivary neurons possess ascending collaterals to the thalamus (Ban and Ohno, 1977; McCrea et al., 1978; Tolbert et al., 1978). This conclusion was mainly based on electrophysiological studies of recordings in the interpositus nuclei and employing antidromic stimulation and collision experiments from thalamus and IO. However, it should be noted that some PIN neurons, especially located in its medial part and within the ICG, project to the contralateral upper cervical cord and course just dorsal to the IO (Bharos et al., 1981; Buisseret-Delmas et al., 1998; Teune et al., 2000). In addition, a major projection from the LCN to the contralateral ventromedial medulla may, in addition, provide projections to the thalamus (Tolbert et al., 1980; Bharos et al., 1981; Teune et al., 2000).

In conclusion, despite the possibility that a very small proportion of the nucleo-olivary neurons may collateralize to the NRTP, we propose that this population of, GABAergic, cerebellar nuclear projection neurons is unique in its projection to the IO. Yet, a definite evaluation of this assertion has to await cell type specific tracing or reconstruction of individual axons.

#### Other nucleo-bulbar projection neurons

Several anatomical studies, including the present data, have shown that many of the medium- and large-sized, excitatory projection cells of the CN project to two or more different brain stem areas (Bentivoglio and Kuypers, 1982; Bentivoglio and Molinari, 1986; Gonzalo-Ruiz and Leichnetz, 1987; Lee et al., 1989). CN axons may bifurctate and follow both the crossed descending limb of the scp toward the caudal medulla and upper cervical cord as well as the crossed ascending limb toward the superior colliculus and thalamus (Bentivoglio and Kuypers, 1982). In addition, individual neurons of the lateral AIN and DLH can both enter the crossed ascending limb but also send one or more collaterals to the ipsilateral bulbar reticular formation (Bentivoglio and Molinari, 1986). Collateralization of many neurons located in the LCN and of a few in the AIN to the basilar pontine nuclei and thalamus were demonstrated by Lee et al. (1989). The same authors also describe a collateral projection to the basilar pontine nuclei and the superior colliculus arising from several LCN neurons. Finally, Gonzalo-Ruiz and Leichnetz (1987) observed double-labeled neurons predominantly in the MCN but also in the LCN after double fluorescent tracer injections in combinations of several areas involved in eye movement control such as the superior colliculus, the paraoculomotor region, and the medial pontine reticular formation.

In our study, we found that all CN regions that contained neurons that project to the NRTP as well as regions that project to either RN or PreRN also contain neurons that were double labeled. However, double-labeled neurons were rather sparse within the medial AIN and within the meager overlapping areas within the PIN. In addition, our data indicate that many CN neurons that project to the RN also provide terminals to the PreRN. The occurrence of double-labeled neurons varied between experiments but, in some instances, could amount to more than 50% of the total number of labeled cells in an area where both types of labeling were found (Table 1). For this reason, it seems quite probable that some neurons, especially in the LCN, will project to more than two of these areas. This would be in line with the observation by Shinoda et al. (1988) who noted that cerebellar nuclear axons with terminals within the RN all continued toward the thalamus. It is remarkable, however, that cells that project to the PreRN are more likely to be double labeled from the RN than vice versa. This would imply that many neurons that project to the RN (and/or directly surrounding area such as the pararubral area) will not consider the PreRN area as a target, whereas neurons that project to the PreRN are likely to also target the RN (Table 1, Figure 9). As yet, it seems feasible to suggest that most cerebellar nuclear cells with an axon in the crossed ascending limb of the scp will ultimately project to the thalamus and may or may not have collaterals to one or several other brain stem areas.

### Output profiles of cerebellar modules

The present data should be discussed in relation to the modular organization of the CN, which is based on the strict organizational pattern of longitudinal strips of Purkinje cells which terminate upon a specific part of the CN and which is mimicked by climbing fibers that originate from particular subdivisions of the IO to the same longitudinal strips or zones of Purkinje cells and that, simultaneously, provide a collateral projection to the CN target nucleus of these strips (Voogd and Bigaré, 1980; Sugihara and Shinoda, 2004, 2007; Ruigrok, 2011; Voogd et al., 2013). In this way, the output of the cerebellar cortex is organized as a series of discrete, parallel olivo-cortico-nuclear modules. As such, the MCN is considered the output nucleus of the A1 module, whereas the lateral extension of the A zone, representing the cortical constituent of the A2 module, specifically outputs by way of the DLP. The B module, mostly present in the lateral vermis of the anterior lobe, targets the LVN. The thin vermal X zone, interspersed between the A1 and B zones targets to the ICG, while the medial PIN is considered to be the output station of the CX zone. The C1, C2, and C3 zones of the intermediate cortex target AIN, PIN, and AIN, respectively. Projections of the D1 and D2 zones are directed to the vLCN and the dLCN, respectively. Finally, the D0 zone, intercalated between D1 and D2, selects the DLH as output station (for review see Voogd et al., 2013).

Figure 9B summarizes the results with respect to the modular organization of the rat cerebellum. Basically, the MCN in the rat can be divided into a caudal, a rostromedial and a rostrolateral subdivisions. The DLP, in addition, may be considered as a distinct MCN subnucleus. The difference in brainstem projections originating from these four MCN target areas is remarkable (Teune et al., 2000) and may be related to the presence of subzones restricted to certain cerebellar lobules (Voogd et al., 1996; Sugihara and Shinoda, 2004). Here, due to the virtual absent retrograde labeling in the rostral parts of the MCN, the rostromedial and rostrolateral parts will not be further considered. The caudal part of the MCN, which receives Purkinje cell axons from vermal lobules VIb,c—IX, including the vermal visual area (Voogd and Barmack, 2006; Voogd et al., 2013), contains many neurons with projections to the NRTP but also targets the prerubral area. In addition, neurons in this MCN subdivision have been shown to project to the thalamus, the superior colliculus, the basilar pontine nuclei, the medullary reticular formation and the cord and to several preoculomotor nuclei (Bentivoglio and Kuypers, 1982; Gonzalo-Ruiz and Leichnetz, 1987; Lee et al., 1989). Many of these projections arise as branches of the same neurons. Finally, the DLP gives rise to a major, crossed, projection to the bulbar reticular formation, which partly branches to the thalamus (Bentivoglio and Kuypers, 1982), but not to the areas investigated in this study (cf. Teune et al., 2000).

The ICG (X-module) possesses diverging projections to the RN, PreRN and, to a lesser degree, NRTP regions. However, its most typical feature is found in its projections to the medial medullary reticular formation and, especially, to the spinal cord with collateralizations to the thalamus (Bentivoglio and Kuypers, 1982; Buisseret-Delmas et al., 1998; Teune et al., 2000).

The LVN (B-module) was not included in our analysis as it at the origin of the lateral vestibulospinal tract and does not seem to participate in any of the ascending projections (Ruigrok, 2013). However, it does contain a population of small cells that project to the dorsal fold of the DAO (Ruigrok and Voogd, 1990).

The zonal targeting of the interposed nuclei in the rat has been subject of some controversy. Here, we essentially adhere to the description offered by Voogd et al. (2013). The medial AIN (receiving mostly input from the C1 and, to a lesser degree, from the C3 zones), apart from the non-collateralizing projections to the IO, targets both RN and PreRN with many neurons collateralizing to both regions. More scant projections are directed to the NRTP. The lateral AIN (also receiving input from the C1 and C3 zones) targets all investigated areas. Collateralization is especially prominent between NRTP and RN. Also within the PIN, classically designated as target of the C2 zone, a division between its rostromedial and caudolateral aspect seems to be relevant. The rostromedial part possesses features similar to the neurons of the ICG with respect to their projections to the cervical cord and thalamus (Bentivoglio and Kuypers, 1982). This part of the PIN is now regarded as target area of the CX zone, which is interspersed between C1 and C2 proper (Sugihara and Shinoda, 2007). However, the rostromedial PIN also gives rise to projections to the RN and, to a lesser extent, the PreRN. These projections also arise from the caudolateral PIN, with many neurons collateralizing to both areas. In addition, collateralizing projections from the lateral PIN have been described to the contralateral thalamus and superior colliculus (Bentivoglio and Kuypers, 1982). Both PIN regions, however, do not provide a significant projection to the NRTP.

The DLH, interspersed between lateral AIN and dLCN, functions as the output nucleus of D0 zone (referred to as Y-zone in Voogd et al., 2013). Like the lateral AIN, the DLH is characterized by branched projections to the ipsilateral lateral medulla oblongata and the thalamus (Bentivoglio and Molinari, 1986). However, neurons in this area may also collateralize to RN and NRTP, and, more sparsely, to the PreRN. In addition, the DLH also issues projections to oculomotor related areas (Gonzalo-Ruiz and Leichnetz, 1987).

The output of the vLCN (D1-module) and the dLCN (D2-module) is directed to all four studied areas. Apart from the projections to the IO, prominent collateralization was observed to the RN (pararubral and parvicellular parts), PreRN and NRTP regions. Collateralizing projections from the LCN were also noted to the superior colliculus, the thalamus, the medial reticular formation, the basilar pontine nuclei and several oculomotor related nuclei (Bentivoglio and Kuypers, 1982; Gonzalo-Ruiz and Leichnetz, 1987; Lee et al., 1989). Indeed, it would appear that extensive collateralization to large variety of brainstem structures is a specific feature of the output nucleus of the D-zone.

### Functional considerations

The cerebellum is suggested to be involved in a great variety of functions which effect the coordination, adaptation, timing, and learning of motor programs as well as of cognitive, emotional and visceral functions (Nisimaru et al., 1991, 2013; Schmahmann and Caplan, 2006; Watson et al., 2013). Nevertheless, cerebellar function is thought to involve a characteristic operation that is performed across various, structurally homogeneous components (Bloedel, 1992; Apps and Garwicz, 2005; Ito, 2006). Our study provides information on the level and organization of cerebellar control on motor behavior, through the RN, the PreRN, the NRTP, and the IO. The observation that from a particular cerebellar nuclear region projections to several brainstem regions may originate, indicates that a particular cerebellar module influences these various brainstem areas simultaneously. Although the influence on the IO originates from a distinct neuronal population, we show that these neurons are completely intermingled with the medium- and large-sized relay cells of the CN (Teune et al., 1995) and are likely to be influenced simultaneously the same Purkinje cells (De Zeeuw and Berrebi, 1995; Teune et al., 1998). However, as yet, it is not known to what extent they respond differently to their input in a physiological situation (e.g., see Hoebeek et al., 2011; Uusisaari and De Schutter, 2011).

The projections to the NRTP should be viewed with respect to its role as major supplier of cerebellar mossy fibers with a distinct collateral input to the CN. The classic electrophysiological studies of Tsukahara et al. (1983) showed that a two-neuron excitatory loop exists between the nucleus interpositus of the cat cerebellum and the NRTP. Our findings on the occurrence of neurons with projections to the RN or PreRN to the NRTP in parts of the CN, both extend and qualify the existence of these two-neuron excitatory loops in the rat. When our retrograde labeling experiments are compared to the results of an anterograde tracing study of the projections from the NRTP and basilar pontine nuclei (Mihailoff, 1993), it appears that closed loops may be present between the NRTP and the lateral portion of the AIN, the LCN, and the caudal MCN (excluding DLP). It is interesting to note that the NRTP loop is absent in medial AIN, a region that has been implicated in the control of the ipsilateral hindlimb (Atkins and Apps, 1997). In contrast, the PIN receives input from the NRTP but does not reciprocate it. The obvious absence of major afferent and efferent connections between the medial AIN and medial PIN with the NRTP, may be compensated by a three neuron-loop, between the RN, lateral reticular nucleus and the medial parts of the interposed nuclei (Tsukahara et al., 1983; Ruigrok and Cella, 1995; Ruigrok et al., 1995a). The lateral reticular nucleus may also be involved in reciprocal connections to the MCN (Ruigrok et al., 1995a), while reciprocal projections also seem to exist between the interposed nuclei and the RN (Huisman et al., 1983). The collateral projections of the loops to the RN and the prerubral area are likely to proceed to the thalamus (Shinoda et al., 1988). Presently, no information seems to be available to determine whether or not the ascending collaterals from these loops also terminate in other target CN areas, such as the superior colliculus, oculomotor-related areas and the reticular formation.

The prerubral area may be particularly involved in establishing a 3-neuron excitatory loop between the CN and the IO. Excitatory connections between the interposed nuclei, the nucleus of Darkschewitsch (one of the nuclei in the prerubral area), and the inferior olivary nuclei have been established in the cat with both anatomical and electrophysiological techniques (Ruigrok et al., 1990; De Zeeuw and Ruigrok, 1994; Ruigrok and Voogd, 1995). The olivary nuclei, in particular the principal and MAOs, are involved in cerebello-midbrain-olivo-cerebellar circuitry, which, in man, is known as the triangle of Guillain-Mollaret (Sarnat et al., 2013). As climbing fiber collaterals provide an excitatory collateral projection to the PIN and LCN (Kitai et al., 1977; Ruigrok, 1997; Hoebeek et al., 2011). The function of these excitatory, CN-prerubral-IO-CN circuits, which in primates is more substantiated in a CN-parvicellular RN-IO-CN circuit, is not well understood (Hoebeek et al., 2011). Interactions of the corticospinal system and cerebellar output at the level of the parvicellular RN/PreRN (Pardoe et al., 2004) may be essential for motor learning.

At the level of the IO the excitatory cerebello-midbrain-olivary-cerebellar circuit interacts with the GABAergic nucleo-olivary pathway (Ruigrok and Voogd, 1995; Best and Regehr, 2009; Bazzigaluppi et al., 2012). Since this projection to the IO does not appear to possess major diverging projections (if any at all) to extra-olivary regions, the information carried by this pathway may be completely independent from the information to other brainstem areas. However, as single Purkinje cells may simultaneously influence nucleo-olivary as well as other CN relay neurons (De Zeeuw and Berrebi, 1995; Teune et al., 1998), activity patterns in both CN cell types may be well correlated and explain why, during movement, the excitability of the IO appears to be reduced (e.g., Horn et al., 1996). In addition, the nucleo-olivary pathway appears to be involved in regulation of the electrotonic coupling between olivary cells, thus regulating its oscillatory properties which may be important for synchronicity and timing of olivary activity (Best and Regehr, 2009; Bazzigaluppi et al., 2012). This may be an important feature for selection, timing and learning of motor functions executed by the cerebellum (Welsh et al., 1995; Van Der Giessen et al., 2008; Llinas, 2009).

### Conflict of interest statement

The authors declare that the research was conducted in the absence of any commercial or financial relationships that could be construed as a potential conflict of interest.
